# A cross-species whole genome siRNA screen in suspension-cultured Chinese hamster ovary cells identifies novel engineering targets

**DOI:** 10.1038/s41598-019-45159-2

**Published:** 2019-06-18

**Authors:** Gerald Klanert, Daniel J. Fernandez, Marcus Weinguny, Peter Eisenhut, Eugen Bühler, Michael Melcher, Steven A. Titus, Andreas B. Diendorfer, Elisabeth Gludovacz, Vaibhav Jadhav, Su Xiao, Beate Stern, Madhu Lal, Joseph Shiloach, Nicole Borth

**Affiliations:** 1 Austrian Centre of Industrial Biotechnology, Graz, Austria; 2 University of Natural Resources and Life Sciences, Vienna, Austria; 3 Division of Preclinical Innovation, NCATS, NIH, Rockville, MD USA; 4 Medical University of Vienna, Vienna, Austria; 5 Biotechnology Core Laboratory, NIDDK, NIH, Bethesda, MD USA; 6 Department of Biomedicine, University of Bergen, Bergen, Norway; 7 UniTargetingResearch AS, Bergen, Norway

**Keywords:** Biotechnology, Cell biology

## Abstract

High-throughput siRNA screens were only recently applied to cell factories to identify novel engineering targets which are able to boost cells towards desired phenotypes. While siRNA libraries exist for model organisms such as mice, no CHO-specific library is publicly available, hindering the application of this technique to CHO cells. The optimization of these cells is of special interest, as they are the main host for the production of therapeutic proteins. Here, we performed a cross-species approach by applying a mouse whole-genome siRNA library to CHO cells, optimized the protocol for suspension cultured cells, as this is the industrial practice for CHO cells, and developed an *in silico* method to identify functioning siRNAs, which also revealed the limitations of using cross-species libraries. With this method, we were able to identify several genes that, upon knockdown, enhanced the total productivity in the primary screen. A second screen validated two of these genes, *Rad21* and *Chd4*, whose knockdown was tested in additional CHO cell lines, confirming the induced high productivity phenotype, but also demonstrating the cell line/clone specificity of engineering effects.

## Introduction

RNA interference (RNAi), which protects cells from parasitic nucleic acid sequences, is a biological mechanism that uses double-stranded RNAs (dsRNAs) to silence genes post-transcriptionally. Since the elucidation of its mechanism^1^, it has been applied as a molecular tool which uses small interfering RNAs (siRNAs) to silence sequence-specific genes precisely. These short dsRNAs with a length of 21–25 nucleotides (nt) and 2 nt 3’ overhangs, can be directly introduced into cells. One of the two strands, the guide strand, is incorporated into the RNA-induced silencing complex (RISC), while the other strand, the passenger strand, is degraded, depending on the thermodynamic asymmetry of the duplex^2^. The integrated strand then directs the RISC to reverse complementary mRNAs, leading to cleavage and degradation of the target sequences. In some cases, siRNAs can also downregulate non-targeted mRNAs, so-called off-targets^3^.

Mammalian cells, and especially Chinese Hamster Ovary (CHO) cells, are used to produce therapeutic proteins for human application^4^. One advantage of CHO cells over other mammalian cell lines is their ability to produce human-like glycosylation without being susceptible to most human viruses^5^. The downregulation of specific disadvantageous genes, mainly pro-apoptotic effectors, enzymes that produce unwanted glycosylation, and metabolic genes, with the help of siRNAs, has been applied to CHO cells to enhance culture performance, which often lead to higher titers and improved product quality, as reviewed in^6^. While this approach is based on prior biological knowledge or target identification, restricting the field of application, a whole genome screen can identify previously unknown targets which were not considered as engineering candidates before^7^. With large-scale siRNA screens being implemented more than 15 years ago^8^, sophisticated analysis methods^9^ have been developed and siRNA libraries targeting all coding genes (whole genome) are commercially available for different organisms. So far the application of such siRNA screens to CHO cells has been hampered by the lack of a CHO specific library, which is primarily due to the fact that genome and corresponding transcriptome sequences for CHO or the originating Chinese hamster were released at much later dates compared to other organisms^5,10–14^.

For this reason we here present the first high-throughput cross-species whole genome siRNA screen performed with suspension CHO cells in protein free medium, producing and secreting eGFP as a model product that can be easily analyzed in the screen. As the closest related species to *Cricetulus griseus*, a commercially available mouse siRNA library was used. A method to evaluate the functionality of the mouse siRNAs in CHO cells was developed, based on sequence alignment to published CHO transcriptomes and the ability to induce a low-viability phenotype upon knockdown of proteasome-associated genes, as proteasome inhibition is known to induce apoptosis and frequently leads to cell death^15^. A secondary screen was conducted to confirm genes with a positive impact on the productivity identified in the primary screen. From this, two genes, *Rad21* and *Chd4*, were identified as true positive hits, and subsequently knocked down in different CHO producer cell lines to assess whether their effect as engineering targets is generally applicable across different CHO cell lines and strains.

## Material and Methods

### Cell cultivation

All cell lines were routinely cultivated in shaker flasks (Corning, USA) in CD CHO media (Thermo Fisher Scientific, USA) with supplements as described below, and incubated in suspension at 37 °C in humidified air. Cells were split every 3–4 days.

CHO-K1 cells (ECACC-CCL61) were in-house adapted to serum-free and suspension growth^16^. They harbor a randomly and stably integrated *eGFP* under the control of a CMV promoter and an N-terminal signal peptide guiding secretion of eGFP (Patent No.: EP 1639111 A2). Cells were supplemented with 8 mM L-Glutamine (Merck KGaA, Germany), 1:500 Anti-clumping agent (Thermo Fisher Scientific) and 300 µg/ml Hygromycin B Gold (Invivogen, USA). The cells (K1-eGFP) were shaken at 5% CO_2_ and at 130 rpm.

CHO-K1 cells (ECACC-CCL61), adapted in-house as mentioned above, expressing human diamine oxidase fused to the Fc-region of IgG (Fc-DAO), were supplemented with 8 mM L-Glutamine, 1:500 Anti-clumping agent and 10 µg/ml Blasticidine (Invivogen), and were incubated at 7% CO_2_, and 140 rpm with a 12.5 mm shaking diameter (now called: K1-DAO). The cell line was generated by using Recombinase-Mediated Cassette Exchange in the same way as the recombinant human diamine oxidase (rhDAO) cell line published in^17^.

CHO-S cells (Thermo Fisher Scientific), stably producing Trastuzumab, were generated by random integration of two plasmids. One plasmid encoded the Trastuzumab light chain and a dihydrofolate reductase (DHFR) gene, the second plasmid encoded the Trastuzumab heavy chain and a neomycin resistance gene. Cells were selected by the addition of 0.7 mg/ml G418 (Invivogen) and 400 nM Methotrexate (MTX) (Merck KGaA). After recovery, cells were sorted for high Trastuzumab secretion (top 1%) four consecutive times into medium containing 800 nM MTX using cold capture^18^ and a fluorescence-activated cell sorter. For this procedure, cells were stained at 4 °C with an anti-human IgG (gamma-chain specific) R-Phycoerythrin antibody produced in goat (1:20 diluted, P9170, Sigma). Afterwards, cells were subcloned twice and screened for high product titers and stability. The resulting cell line (S-HERC) was supplemented with 8 mM L-Glutamine, 1:500 Anti-clumping agent, 800 nM Methotrexate (MTX) (Merck KGaA) and 0.7 mg/ml G418. Cells were incubated at 7% CO_2_, and 140 rpm with a 12.5 mm shaking diameter.

CHO-DUKXB11 (ATCC® CRL-9096), stably producing an Erythropoietin-Fc (Epo-Fc) fusion protein^19^, were supplemented with 8 mM L-Glutamine and 0.36 µM MTX (DUKXB11-EPO-8). A derivative cell line, adapted to growth without glutamine^20^ was supplemented only with 0.36 µM MTX (DUKXB11-EPO-0). Both cell lines were incubated at 7% CO_2_ and 140 rpm with a 12.5 mm shaking diameter.

CHO-K1 cells (ECACC-CCL61), adapted as mentioned above, were supplemented with 8 mM L-Glutamine and 1:500 Anti-clumping agent and incubated at 7% CO_2_ and 140 rpm with a 12.5 mm shaking diameter.

### Screening assay development

A non-targeting control (Silencer® Select Negative Control No. 2 siRNA, Thermo Fisher Scientific) and a positive control (AllStars Mm/Rn Cell Death Control siRNA, QIAGEN, Germany) were spotted (2 µl of 400 nM stocks) into 384 well plates (Cat. No.: 3707, Corning). Nuclease-free water was used as mock control. Different amounts of RNAiMAX (0–0.6 µl per well; Lipofectamine® RNAiMAX Transfection Reagent, Thermo Fisher Scientific) were diluted in screening media (20 µl per well; CD CHO supplemented with 8 mM L-Glutamine), incubated for at least 10 min at room temperature (RT), and added to the wells. K1-eGFP in exponential growth phase (day 3 post splitting, between 1.5E6 and 3.5E6 cells/ml, above 90% viability) were spun down (200 × g, 8 min) and re-suspended in screening media. 20 µl of the cell suspension, containing varying cell numbers (2500–5500 cells) were seeded into the wells already containing the siRNA-lipid complexes. The plates were incubated at 37 °C, 5% CO_2_ and 95% humidity. After three days of incubation, 30 µl of CTG (CellTiter-Glo® Luminescent Cell Viability Assay, Promega, USA) containing 0.05% trypsin-EDTA (Gibco™ Trypsin-EDTA (0.05%), Thermo Fisher Scientific) were added to the wells and incubated for 20 min at RT. The luminescent readouts were collected by the EnVision multilabel reader (PerkinElmer, USA).

The above protocol was optimized by varying incubation time (2–4 days) and media supplements (addition of Anti-clumping agent (1:500) or Hygromycin B Gold (300 µg/ml)). Furthermore, four different non-targeting siRNAs (Silencer® Select Negative Control No. 1 siRNA, Thermo Fisher Scientific; Silencer® Select Negative Control No. 2 siRNA, Thermo Fisher Scientific; AllStars Negative Control siRNA, QIAGEN; Silencer® Negative Control No. 5 siRNA, Thermo Fisher Scientific) were tested under the same conditions. Statistical differences between the non-targeting siRNAs were analyzed with the statistical software R version 3.4.0^21^. An Anderson-Darling test, as included in the R package nortest^22^, was applied to the luminescence readings to test for normal distribution, and a Levene-test, included in the package R package car^23^ was executed to test for equal variances. As normality was not proven, a Kruskal-Wallis test was applied, and pairwise Wilcoxon rank sum tests with Bonferroni-adjusted p-values were used as *post hoc* test.

### Primary screen

The primary RNAi screen was conducted using the Ambion Silencer® Mouse Genome siRNA Library Version 3, which consists of three unique, non-overlapping, non-pooled siRNAs per gene target. siRNA reagents (2 µl of a 400 nM stock) were stamped into 384 well microplates (black, clear flat bottom, Cat. No.: 781091, Greiner Bio-One, Austria) using a Velocity11 VPrep liquid handling system (Agilent, USA) integrated into a BioCel robotic platform (Agilent) in columns 1–22, leaving columns 23–24 empty for negative (Silencer® Select Negative Control No. 2 siRNA, Thermo Fisher Scientific) and positive (AllStars Mm/Rn Cell Death Control siRNA) controls, respectively. RNAiMAX (0.2 µL; Invitrogen) was added in 20 µL screening media to wells using a Matrix WellMate and Microplate Stacker (Thermo Fisher Scientific). Plates were incubated for 45 minutes at room temperature to allow for formation of siRNA-lipid complexes. Cells were seeded at a density of 2500 cells/well in 20 µL screening media and cultured for 4 days at 37 °C, 5% CO_2_ and 95% humidity. Then, intracellular GFP-intensity was detected using a laser cytometer (acumen® Cellista laser scanning imaging cytometer, TTP Labtech, UK). Subsequently, 30 µl of CTG were added per well, incubated for 20 min at RT, and luminescent reads were collected via the EnVision multilabel reader (Supplementary Table 3).

### Secondary screen

Genes with the highest median seed-corrected total intracellular fluorescence intensity from the primary screen were chosen and three independent siRNAs (QIAGEN) against these genes were selected from the available library and screened as above (Supplementary Table 10), with the modification that each siRNA was screened three times. In a few cases, the siRNA sequence was the same as in the primary screening library, in which case it was eliminated from the data set as it would not be an independent data point (see Supplementary Table 11 for independent siRNAs). Genes with a median total fluorescence higher than the median total fluorescence of the primary screen plus 3x the median absolute deviation were selected for further downstream, orthogonal validation.

### Data processing

The data was processed with the statistical software R version 3.4.0^21^. Both luminescence and intracellular fluorescence intensities were normalized to the negative control by dividing the signals of each sample well through the median signal of the negative control wells per plate, and the normalized results of all but 68 siRNAs, whose respective target genes were withdrawn by NCBI (Supplementary Tables 4 and 11), were deposited at the PubChem open chemistry database (https://pubchem.ncbi.nlm.nih.gov/bioassay/1259405). As for the secondary screen, each siRNA sequence was screened thrice, the median of the three replicates was used for further processing. In addition, the negative control was used to assess edge well effects in both screens. For this, the median and standard deviation was calculated for each position within the negative control column over all plates per screen.

Off-target effects of sample wells were corrected by applying a common seed-based^24^ correction method. In brief, all siRNA sequences of the primary screen were grouped by their seed sequence (2^nd^ to 7^th^ nucleotide of the siRNA sequence), and correction factors were assigned by calculating the difference between the median of each seed sequence and the overall median of the primary screen for luminescence and intracellular fluorescence intensities. If an siRNA exhibits an effect larger than the seed-based median and in the same direction, the effect is corrected by subtracting the correction factor from the effect. If an siRNA exhibits an effect smaller than the seed-based median and in the same direction, the effect is set to the overall median. If an siRNA exhibits an effect in the opposite direction than the seed-based median, it remains unchanged. Results from both screens were corrected by the correction factors calculated from the primary screen.

The siRNA sequences were aligned to three publicly available CHO transcriptomes (RefSeq-1, RefSeq-2 (RefSeq v80), Public-1^25^) and two in-house transcriptomes^26^ (Supplementary Table 1) with the help of bowtie version 1.2.2^27^. The data was then further processed with the statistical software R version 3.4.0. All siRNAs with ≤1 mismatch to at least one transcript in the right orientation (reverse complementary) were kept for further processing. Gene information for each transcript was retrieved using data obtained from the same platforms as the transcriptomes (ftp://ftp.ncbi.nlm.nih.gov/gene/DATA/gene2accession.gz, retrieved on 31.01.2017 and filtered for the taxonomy 10029 (*Cricetulus griseus*), and https://gendbe.computational.bio.uni-giessen.de/cho.html, SAMS – functional annotation (CSV), retrieved on 31.01.2017) and siRNAs were associated with the corresponding genes.

The effect of siRNAs with one mismatch against a transcript was evaluated by correlating the median of luminescence readings of siRNAs from the primary screen targeting proteasome-associated genes with or without a mismatch. Proteasome-associated genes of CHO were retrieved from the Kyoto Encyclopedia of Genes and Genomes^28^ (cge03050 (Release 90.0) was retrieved on 29.04.2019) with the help of the R-package KEGGprofile version 1.22.0^29^.

### siRNA validation

siRNAs (Eurofins, Luxembourg) were transfected into CHO-K1 cells using the Neon® transfection system (Thermo Fisher Scientific) with the Neon® transfection system 100 µl kit (Thermo Fisher Scientific) according to the manufacturer’s protocol. In brief, 5E6 cells were spun down (170 × g, 8 min) and resuspended in 100 µl buffer R. After the addition of 300 pmol of siRNAs, cells were shocked by applying one pulse with 1700 V and 20 ms. A mock transfection and a non-targeting siRNA (AllStars Negative Control siRNA, QIAGEN) were included as controls. Cells were allowed to recover in 10 ml media in Tubespin® bioreactors 50 (Techno Plastic products, Switzerland) for at least 2 hours post transfection without shaking at 37 °C, humidified air and 7% CO_2_. Afterwards, cultures were shaken at 250 rpm with a 12.5 mm shaking diameter.

### RNA isolation

Total RNA was isolated using TRI® reagent (Merck KGaA) according to the manufacturer’s protocol. In brief, 3E6 cells were harvested on day 2 post transfection, spun down (400 × g, 6 min) and resuspended in 1 ml TRI® reagent. 200 µl of Chloroform were added, the samples were mixed and centrifuged at 4 °C for phase separation. 500 µl 2-Propanol were added to the upper, aqueous phase, and samples were centrifuged for RNA precipitation and pelleting. The pellets were washed with 75% Ethanol, air-dried, resuspended in 30 µl nuclease-free water and incubated at 65 °C for 10 min. Quality and quantity of RNA was determined by a NanoDrop^TM^ One UV-Vis Spectrophotometer (Thermo Fisher Scientific). Only RNA samples with a 260/280 and a 260/230 ratio above 1.8 were used.

### Gene expression quantitation

800 ng of total RNA were reverse-transcribed using the High Capacity cDNA Reverse Transcription Kit (Thermo Fisher Scientific) with RNase Inhibitor according to the manufacturer’s protocol. A reverse transcription control (RTC) of the respective mock transfection, lacking the reverse transcriptase, was included. The resulting cDNAs and RTCs were 1:4 diluted with nuclease-free water. RT-qPCR was performed in quadruplets for each cDNA with the SensiFAST^TM^ SYBR® Hi-ROX Kit (Bioline Reagents, UK) according to the manufacturer’s protocol, on the Rotor-Gene Q (QIAGEN). Non-template controls and RTCs were included in duplets for each primer pair (Supplementary Table 2). Assays were downscaled to 10 µl per reaction. Gene expression levels were relatively quantified with the 2^−ΔΔCT^ method^30^ against *Gapdh*. Fold changes were related to the mock transfected control.

### Batch culture

Equimolar mixes of 3 siRNAs targeting each gene were transfected into two replicates of S-HERC, DUKXB11-EPO-8, DUKXB11-EPO-0 and K1-DAO each, including two mock transfections as control using the same conditions as in section 2.6. Cells were allowed to recover in 20 ml media in Tubespin® bioreactors 50 for at least 2 hours post transfection at 37 °C, humidified air and 7% CO_2_, but without shaking. Afterwards, cultures were shaken at 250 rpm with a 12.5 mm shaking diameter. Viable cell density (VCD), viability and viable cell diameter were measured daily by the Vi-Cell XR (Beckman Coulter Inc.). Culture supernatants were obtained on a daily basis by spinning down 300 µl of the cultures (200 × g, 5 min) and collecting the supernatants.

### Product quantification

Product concentrations were determined with the Octet® QKe (Pall Corp., USA), equipped with Dip and Read^TM^ Protein A Biosensors (Pall Corp) according to the manufacturer’s recommendations. Culture supernatants were diluted 1:4 in phosphate-buffered saline +0.1% Tween (pH 7.3) prior to measurement, except for samples taken on day 8 post transfection from DUKXB11-EPO-0, DUKXB11-EPO-8 and K1-DAO, which were 1:8 diluted. Serial dilutions (10 × 1:2 dilutions) of purified Epo-Fc (in-house) or Trastuzumab (BioVision, USA), starting with 37.5 µg/ml for Epo-Fc and 100 µg/ml for Trastuzumab, were included as standards for absolute quantifications. Fc-DAO was quantified relatively, as no Fc-DAO standard was available.

### Culture characterization

All calculations were done with the statistical software R version 3.4.0 and an in-house R-package vicellR version 0.1.9 (in development). The volume per cell was calculated with the assumption that a cell is a perfect sphere:

$${\rm{volume}}\,{\rm{per}}\,{\rm{cell}}[\frac{\mu {m}^{3}}{cell}]=\,\frac{4}{3}\cdot \pi \cdot {(\frac{{\rm{viable}}{\rm{cell}}{\rm{diameter}}[\mu m]}{2})}^{3}$$

The viable cell volumes (VCV) were calculated by multiplying the volume per cell with the VCD.

$${\rm{VCV}}[\frac{m{m}^{3}}{ml}]={\rm{volume}}\,{\rm{per}}\,{\rm{cell}}\cdot {10}^{-9}\cdot {\rm{VCD}}\lfloor \frac{cells}{ml}\rfloor $$

A one-way ANOVA was used to determine significant differences between the mock control and the gene knockdowns at each time point, and the Dunnett’s test, included in the multcomp-package version 1.4.8^31^, was used as *post hoc* test.

Replicates were combined and seed-corrected based on the first measurement of the VCD (or VCV). Pearson’s correlation coefficients were determined for linear correlations between ln-transformed VCD (or VCV) and the culture time, starting from the first time point analyzed (TP01) and including at least 4 time points. For each sample the highest correlation coefficient (r_MAX_) and the time point (TPXY) of its occurrence were determined. The growth rates were calculated as slopes in simple linear regressions of the ln-transformed VCD (or VCV) versus the interval (TP01–TPXY), where the minimum is taken over treatments.

Cumulative viable cell days (CCD_CD_ or CCD_CV_, based on VCD or VCV) were calculated by

$${{\rm{CCD}}}_{{\rm{CD}}}[cells\cdot days]=\sum _{{\rm{i}}=1}^{{\rm{n}}-1}\frac{({{\rm{VCD}}}_{{\rm{i}}+1}-{{\rm{VCD}}}_{{\rm{i}}})\cdot ({{\rm{t}}}_{{\rm{i}}+1}-{{\rm{t}}}_{{\rm{i}}})}{(\mathrm{ln}({{\rm{VCD}}}_{{\rm{i}}+1})-{\mathrm{ln}(\mathrm{VCD}}_{{\rm{i}}}))\cdot 24}$$

$${{\rm{CCD}}}_{{\rm{CV}}}[c{m}^{3}\cdot days]=\sum _{{\rm{i}}=1}^{{\rm{n}}-1}\frac{({{\rm{VCV}}}_{{\rm{i}}+1}-{{\rm{VCV}}}_{{\rm{i}}})\cdot {10}^{-3}\cdot ({{\rm{t}}}_{{\rm{i}}+1}-{{\rm{t}}}_{{\rm{i}}})}{(\mathrm{ln}({{\rm{VCV}}}_{{\rm{i}}+1}\cdot {10}^{-3})-{\mathrm{ln}(\mathrm{VCV}}_{{\rm{i}}}\cdot {10}^{-3}))\cdot 24}$$

where t represents the hours post transfection, and n the number of time points analyzed per sample. Replicates were combined and seed-corrected based on the calculated CCD_CD_ (or CCD_CV_) of the second time point. Pearson’s correlation coefficients were determined for linear correlations between CCD_CD_ (or CCD_CV_) and the titers, starting from the second measurement (TP02) and including at least 5 time points. For each sample the highest correlation coefficient (r_MAX_) and the time point of its occurrence (TPXY) was determined. The specific productivities were calculated as slopes in simple linear regressions of the CCD_CD_ (or CCD_CV_) versus the titers for the interval (TP01 – TPXY), where the minimum is taken over treatments.

All raw data from measurements, transcriptomes, bowtie-alignments, transcript-gene-associations, R-scripts, and the in-house developed R-package vicellR version 0.1.9 are available at figshare (https://figshare.com/s/ee4d2ee4640404e660aa).

## Results

### Assay development

The initial screen optimization revealed that the lowest positive/non-targeting control ratio for lipofection is obtained with 2 µl of a 400 nM siRNA stock solution, 2500 K1-eGFP cells and 0.2 µl RNAiMax (lipofection reagent) per well (Supplementary Fig. 1). With these conditions fixed, the ideal analysis time point was determined and the influence of media supplements used for standard cultivation of cells on lipofection analyzed. Hygromycin B Gold induced lower growth and a higher positive/non-targeting control ratio, while the Anti-clumping agent led to a positive/non-targeting control ratio of around 1, indicating a non-functional lipofection (Supplementary Fig. 2). Therefore, both media supplements were removed during screening. Several non-targeting controls were tested under these optimized conditions, and as there was no significant difference between the Silencer® Select Negative Control No. 2 siRNA and any other tested non-targeting control (all adjuted p-values above 0.05, Supplementary Fig. 3), Silencer® Select Negative Control No. 2 siRNA was chosen for the primary screen.

### Primary screen

The workflow of the two screens and the validating batch cultures is depicted in Supplementary Fig. 4. A whole genome mouse siRNA library consisting of 50168 unique siRNAs targeting 17572 genes was applied to K1-eGFP cells using the above optimized conditions (Supplementary Fig. 5). Each well contained a single siRNA sequence, with 2.85 siRNA sequences per gene on average. A high humidity incubator was used to reduce edge well effects by evaporation, thus no significant spatial effect was visible (Supplementary Fig. 6). Total intracellular fluorescence intensity (specific GFP productivity) and total luminescence after CTG-treatment (viable cell density) were detected for each well, and both readouts were normalized to the non-targeting control included on each plate (Supplementary Table 4). Seed-based correction factors were applied to the intracellular fluorescence and to the luminescence readouts based on all siRNA sequences (Supplementary Table 5), and measurement values were adjusted accordingly.

### Identifying valid siRNAs

**Figure 1:**
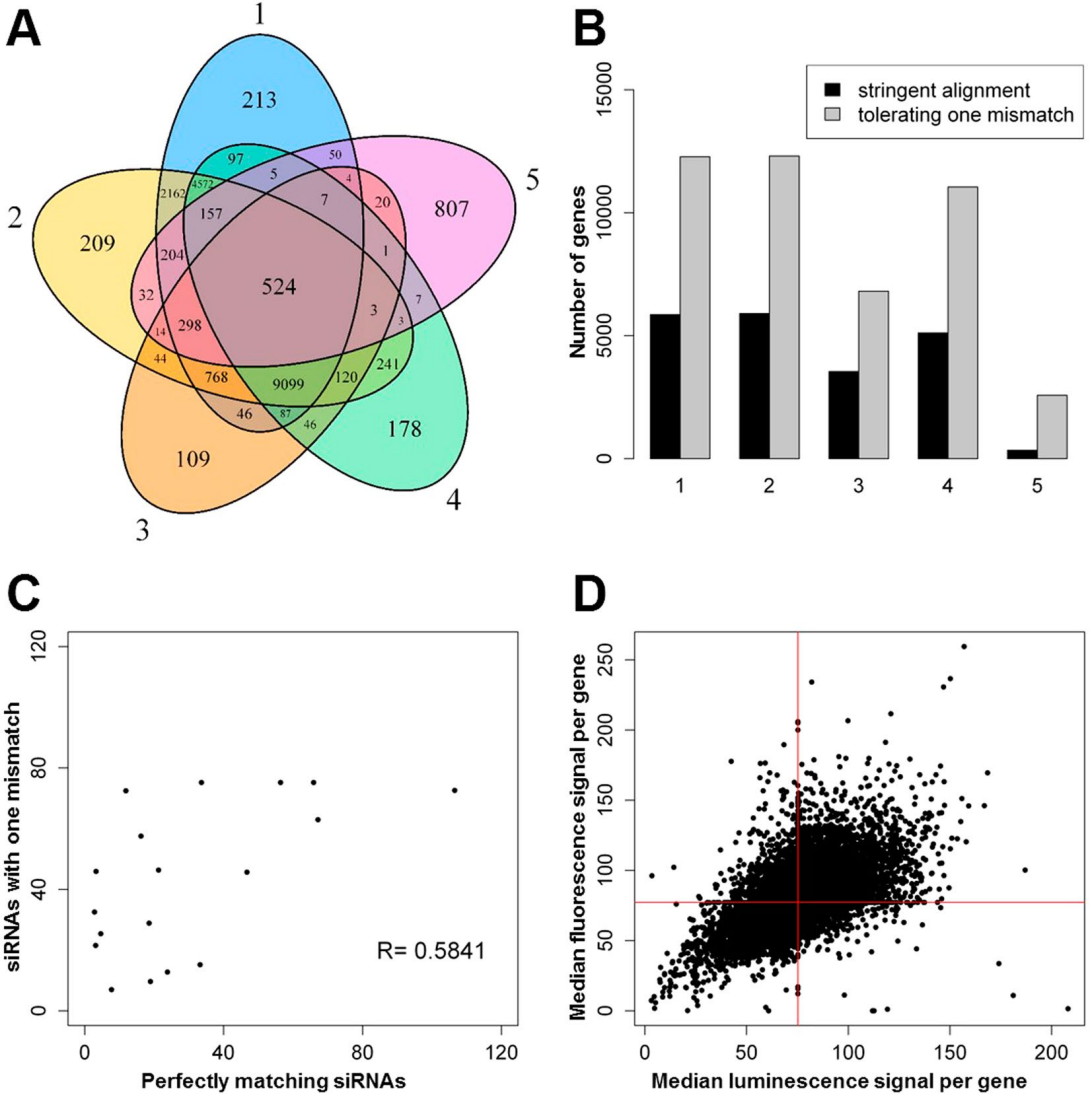
Primary screen. (**A**) siRNA alignment to the 5 CHO transcriptomes (1: RefSeq-1. 2: RefSeq-2. 3: Public-1. 4: In-house-1. 5: In-house-2). Numbers inside the Venn diagram indicate number of siRNAs targeting each transcriptome. (**B**) Genes of each transcriptome targeted by primary screen with stringent alignment or with tolerating one mismatch. (**C**) Median luminescence signals of siRNAs perfectly matching proteasome-associated genes versus median luminescence signals of siRNAs with one mismatch against the same proteasome-associated genes. Each dot represents a proteasome-associated gene. (**D**) Median fluorescence versus median luminescence signal per targeted gene. Red lines: Median of the primary screen.

Out of the 50168 siRNA sequences, 20127 were successfully mapped to at least one transcript in the five CHO transcriptomes with ≤ one mismatch (Fig. 1A). Transcripts were combined according to their respective genes (Fig. 1B, Supplementary Table 6). Gene information was available for all but one transcript (XM_007649951.2). Genes of the different transcriptomes were then grouped based on the same siRNAs targeting the respective gene, leading to 17807 distinguishable CHO genes targeted by this screen with an average of 1.64 siRNAs per gene (Supplementary Table 7). Median values of intracellular fluorescence intensities and of luminescence intensities were calculated per targeted gene (Fig. 1D).

45 proteasome-associated genes were retrieved from KEGG pathway cge03050, with 18 of them targeted by at least one siRNA with a mismatch and one without a mismatch (Supplementary Table 8). The medians of the luminescence signals were correlated for siRNAs with a mismatch against siRNAs without a mismatch (Fig. 1C). A linear correlation coefficient of ~0.58 shows that siRNAs with one mismatch exhibit a tolerably comparable effect to those siRNAs that fully match their target genes. Therefore, all siRNAs with up to 1 mismatch were accepted as specific.

### Gene selection for validation in the secondary screen

**Figure 2:**
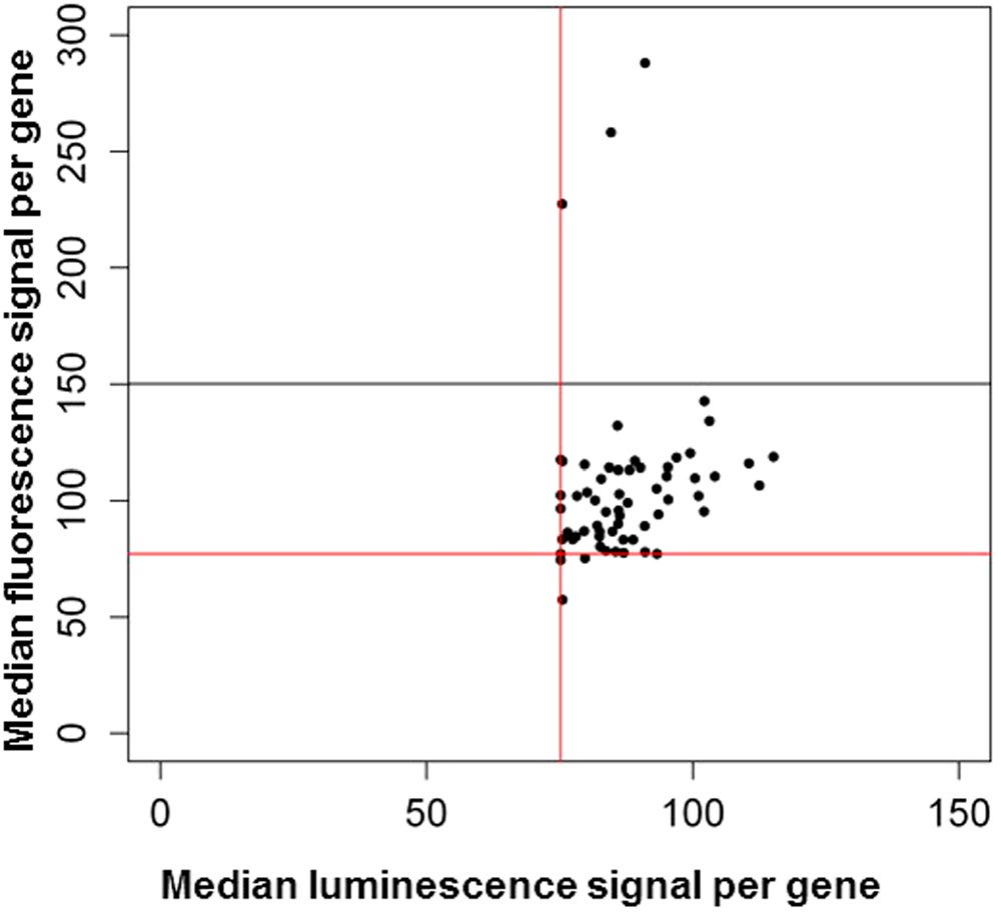
Secondary screen. Median intracellular fluorescence versus median luminescence signal per targeted gene of each gene tested in the secondary screen. Red lines: Median of the primary screen. Black line: Median of the primary screen + 3x Median absolute deviation of the primary screen.

Next, we intended to validate the 135 CHO genes with the highest median intracellular fluorescence intensity (Supplementary Table 9) with new siRNAs, but due to technical issues we were only able to test 58 of these (Supplementary Table 12). Median values of intracellular fluorescence intensities and of luminescence intensities were calculated per targeted gene (Fig. 2). In this smaller screen of only 3 plates, a significant edge well effect is visible (Supplementary Fig. 6). This can result in higher luminescence and fluorescence readouts at the siRNAs present at the edge wells of the plates leading to a skewed data set. As the effect is mostly caused by one of the three plates, and each siRNA was screened thrice on three separate plates, with the median of the three values used for further data processing, we still kept all three plates for the analysis. Three of the genes show a significant increase in total intracellular fluorescence intensity (above median of primary screen +3x Median absolute deviation; Supplementary Table 13). Two of these genes are the *Rad21* gene of different transcriptomes, the third one is the *Chd4* gene.

### Effects of knockdown of Chd4 and Rad21 in other CHO producer cell lines

**Figure 3:**
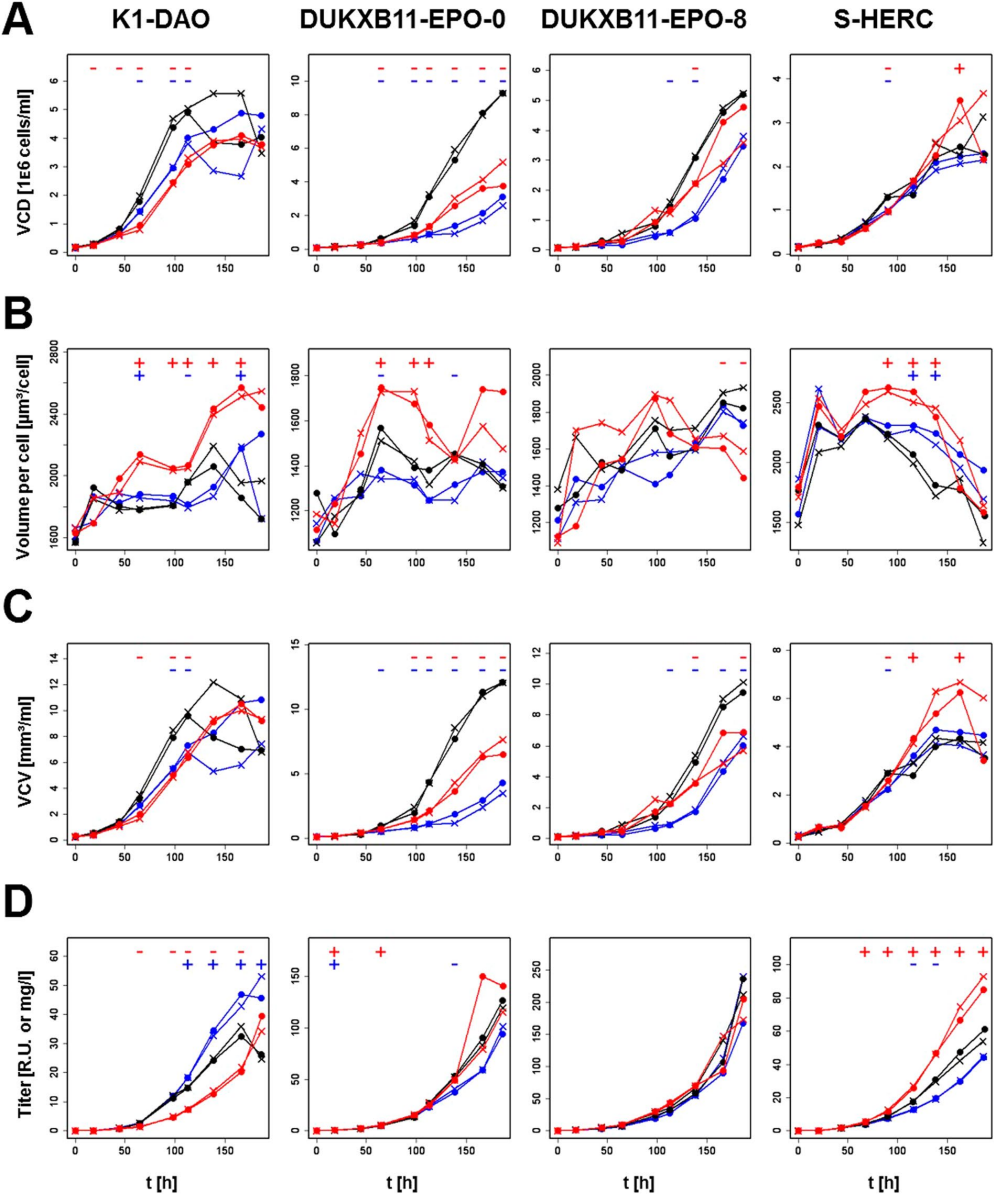
Batch cultivation of producer cell lines. Black lines: Mock control. Red lines: *Rad21*-KD. Blue lines: *Chd4*-KD. X: Replicate 1. Dot: Replicate 2. Minus (−): Significant (p-values < 0.05) downregulation between the respective knockdown and the mock control. Plus (+): Significant (p-values < 0.05) upregulation between the respective knockdown and the mock control at the specific time point. (**A**) VCD over time of the batch cultures. (**B**) Cellular volume over time of the batch cultures. (**C**) VCV over time of the batch cultures. (**D**) Titer over time of the batch cultures.

**Figure 4:**
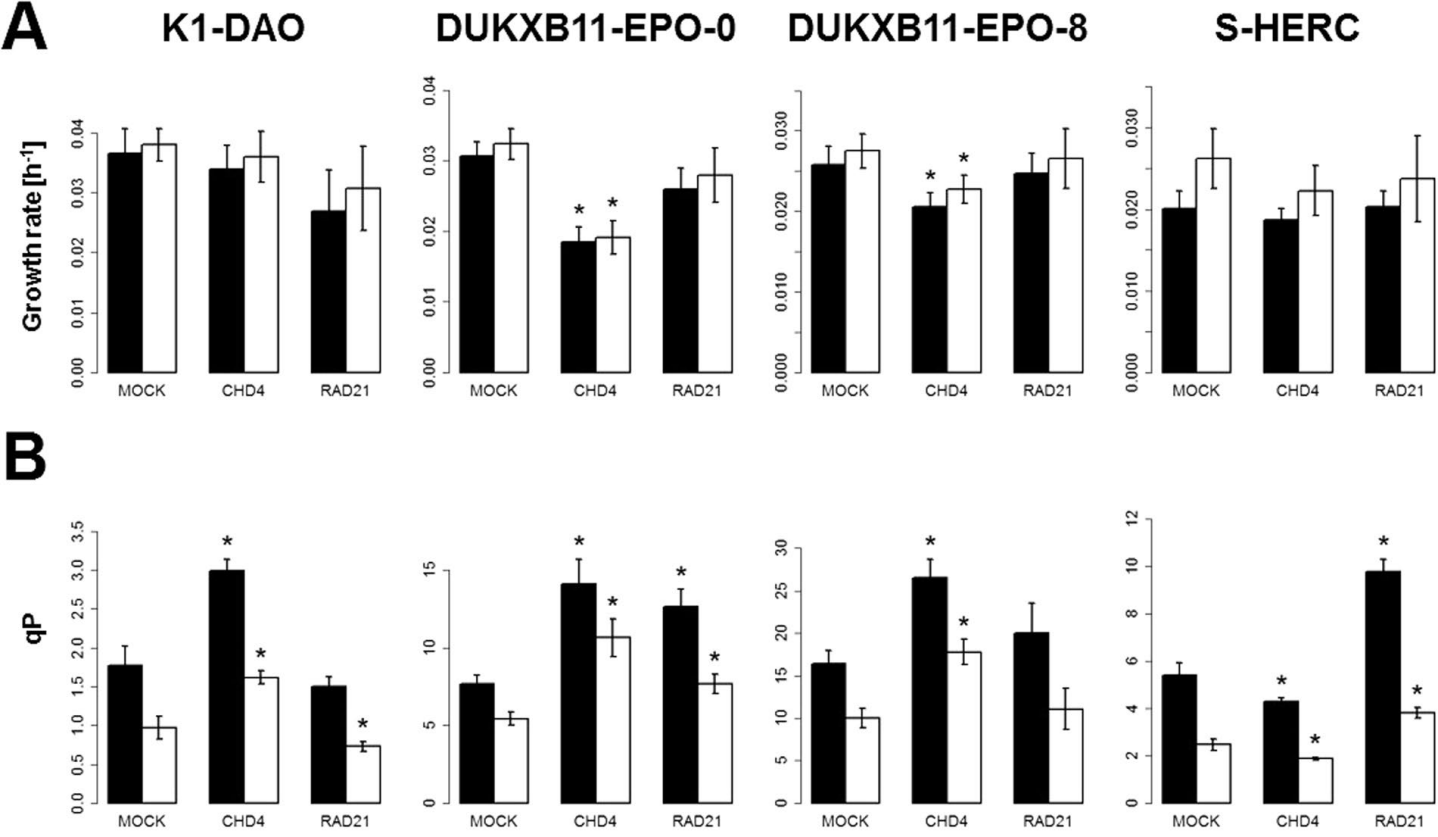
Growth rates and specific productivities of producer cell lines. (**A**) Growth rates of the batch cultures calculated by VCD (black bars) or VCV (white bars). (**B**) QP of the batch cultures calculated by VCD (black bars, pg/(cell*day)) or VCV (white bars, mg/(cm³*day)). Error bars represent 95% confidence interval. *significant change in comparison to the respective mock-sample (p-values < 0.05).

To validate the general applicability of knockdown (KD) of these genes in other production cell lines, the KD efficiencies of all siRNAs which targeted either *Chd4* or *Rad21* in the primary and secondary screening in CHO-K1 host cells were tested. siRNAs targeting *Chd4* achieved a KD between 37 and 82%, and siRNAs targeting *Rad21* were able to knockdown 83 to 94% of the mRNA signal (Supplementary Fig. 7), confirming the on-target effect of these siRNAs. Three of the siRNAs for each gene were then mixed equimolarly (Supplementary Table 14), to reduce off-target effects of individual siRNA species, and delivered into four different producer cell lines (S-HERC, DUKXB11-EPO-8, DUKXB11-EPO-0 and K1-DAO) to evaluate effects on growth rate, cell size, titer and specific productivity (Figs 3 and 4). As the cell size was affected by the knockdowns (KD) (Fig. 3B), both growth rates and specific productivities were calculated based on the VCD and VCV. The KD of at least one of the two genes led to a higher specific productivity in all cell lines, while the KD of the second gene sometimes reduced the specific productivity (Fig. 4B). In some cases the KD also reduced the exponential growth rate, leading to unchanged or reduced titers (Fig. 3 and 4A). Still, the overall titer was increased in two cell lines, once by *Chd4*-KD (K1-DAO) and once by *Rad21*-KD (S-HERC) (Fig. 3D).

## Discussion

To understand gene functionality in suspension grown CHO cells and potential correlations to productivity and growth, a genome wide gene KD screen was conducted as the method of choice for an initial high throughput method with few side effects. An alternative assay would have been to use a knockdown library based on genome editing. The CRISPR-system has been well established in CHO cells, but the knockout efficiencies are often low^32–35^. Thus knockouts are mostly used to generate stable cell lines in combination with additional selection methods to increase efficiencies^35–40^. The CRISPR technology can also be applied with a catalytically inactive Cas9 (dCas9) to knockdown specific genes (CRISPR interference or CRISPRi^41^), but this system needs additional recombinant proteins to be delivered into the cells next to the guide RNA, which could lead to unknown side effects. Thus siRNAs are the simplest way of knocking down genes transiently, as only a small RNA has to be delivered into the cells, which will then use the endogenous cellular machinery to reduce gene expression^2^.

As no CHO-specific whole genome siRNA library was available, a cross-species siRNA screening assay using a mouse siRNA library was conducted. While we eagerly await an siRNA library designed against the CHO genome, the mouse siRNA library had the distinct advantage of being readily commercially available and having many siRNAs that target the CHO transcriptome^42^. To take into account the sequence dissimilarities, a method to validate cross-species siRNAs was developed which is based on the alignment of siRNA sequences to available transcriptomes of CHO. Due to such sequence dissimilarities, only 20127 siRNAs targeting 17807 CHO genes by on average 1.64 siRNAs allowing up to one mismatch per gene could be used for evaluation. The exclusion of ~59% of the siRNAs led to a lower siRNA coverage per gene, which demonstrates the limitations of cross-species screens. Note that the coverage per gene is higher than expected, because an siRNA can target more than one CHO gene. Although our results show that a single mismatch is tolerable in that it still may generate an effect on cell behavior, taken together it is clear that a full genome siRNA set designed specifically for CHO would significantly improve the results obtained, simply by enlarging the group of genes that can be tested and by increasing the coverage per gene.

In this screening approach, only an increase in total intracellular fluorescence as read-out was taken into consideration to identify engineering targets that increase the total yield in culture, as it is known that intracellular product content correlates well with specific productivity^43,44^. However, an increase in the number of cells will also increase the total fluorescence measured in this screen. The validity and importance of running whole genome screens is confirmed by the two genes that were identified in our results, neither of which would intuitively be connected to improved specific productivity in a production cell line. The chromodomain helicase DNA-binding protein 4 (*Chd4*) is a component of the Nucleosome remodeling and deacetylase (NuRD) complex and has nucleosome remodeling activity^45^. The depletion of *Chd4* can help in reactivating epigenetically silenced genes^46^. The second gene, the *Rad21* cohesin complex subunit, is a component of cohesin, a multi-protein complex, which mediates sister chromatid cohesion^47^ and is required for centromere integrity^48^. Also, Rad21 is cleaved by caspases upon apoptotic stimuli^49^, and the C-terminal cleavage product is then able to promote apoptosis^50^. Apart from that, Rad21 also regulates gene expression by enabling the transcriptional insulator ability of CCCTC-binding factor^51^. Both Chd4 and Rad21 are involved in DNA double strand break repair^52,53^, and co-localize on different Alu repeats^54^. How these properties might contribute to increase growth or productivity could only be speculation at the current stage.

Interestingly, the effect of knockdown of these genes varied between different CHO cell lines tested, confirming the known variation between CHO cell line and subclone phenotypes and the sometimes contradictory results of overexpression of a likely engineering target. An example for this is the overexpression of PDI, an ER chaperone that mediates disulfide bond formation, whose overexpression may^55–57^ or may not^58^ improve antibody production. It is possible that the specific transcriptome defines the susceptibility of a cell line for engineering of a given gene. Thus, if a gene that increases specific productivity is lowly expressed in one cell line and highly expressed in another, its overexpression may work in the first and have no effect in the second. Vice-versa, a gene that impairs high production rate may be successfully knocked down in a cell line that expresses it at high level, while little effects are observed in a cell line that has already decreased expression. Thus cell line engineering approaches are likely to be dependent of the overall transcriptome context^59,60^ and may in the future have to be “personalized” to the specific needs of a given cell line. This also implies that such screens should routinely be run with more than one model cell line to ensure identification of all relevant genes. This outcome also confirms the importance of running such a screen in the context of CHO cell lines and recombinant protein production under conditions that are as close to industrial process conditions as possible and thus justifies our effort to develop a screening protocol that uses cells grown in suspension and in a chemically defined, protein free medium.

## Conclusion

Full genome approaches to high throughput screening offer many advantages, such as identification of engineering targets that were not considered before. To take full advantage of the technology, however, a species specific library is required and it is necessary to run a screen with different cell lines and subclones in parallel, to enable identification of all possible targets which may depend on the cell line specific transcriptome context.

The two targets identified in this screen are promising, but will require further studies to understand the underlying mechanism and to identify the optimal transcript level as well as the effect of a full deletion and possible synergistic effects.

## Supplementary information

Supplementary Figures

Supplementary Tables
