# Supplementary material for: A cross-species whole genome siRNA screen in suspension-cultured Chinese hamster ovary cells identifies novel engineering targets
Source: Sci Rep. 2019 Jun 18;9:8689. doi: 10.1038/s41598-019-45159-2 (PMC6582146; doi:10.1038/s41598-019-45159-2)
Supplement: Supplementary file 1 — Supplementary Figures [file 41598_2019_45159_MOESM1_ESM.docx]

**A cross-species whole genome siRNA screen in suspension-cultured Chinese hamster ovary cells identifies novel engineering targets**

Gerald Klanert^a^, Daniel J. Fernandez^c^, Marcus Weinguny^a^, Peter Eisenhut^a^, Eugen Bühler^c^, Michael Melcher^a,e^, Steven A. Titus^c^, Andreas B. Diendorfer^a^, Elisabeth Gludovacz^g^, Vaibhav Jadhav^a^, Su Xiao^b^, Beate Stern^d,f^, Madhu Lal*^c^, Joseph Shiloach*^b^, Nicole Borth*^a,e^

a Austrian Centre of Industrial Biotechnology, Graz, Austria

b Biotechnology Core Laboratory, NIDDK, NIH, Bethesda, MD, USA

c Division of Preclinical Innovation, NCATS, NIH, Rockville, MD, USA

d Department of Biomedicine, University of Bergen, Norway

e University of Natural Resources and Life Sciences, Vienna, Austria

f UniTargetingResearch AS, Bergen, Norway

g Medical University of Vienna

* These authors jointly supervised the work

Email:

madhu.lal@nih.gov (Madhu Lal-Nag)

josephs@niddk.nih.gov (Joseph Shiloach)

nicole.borth@boku.ac.at (Nicole Borth)

**
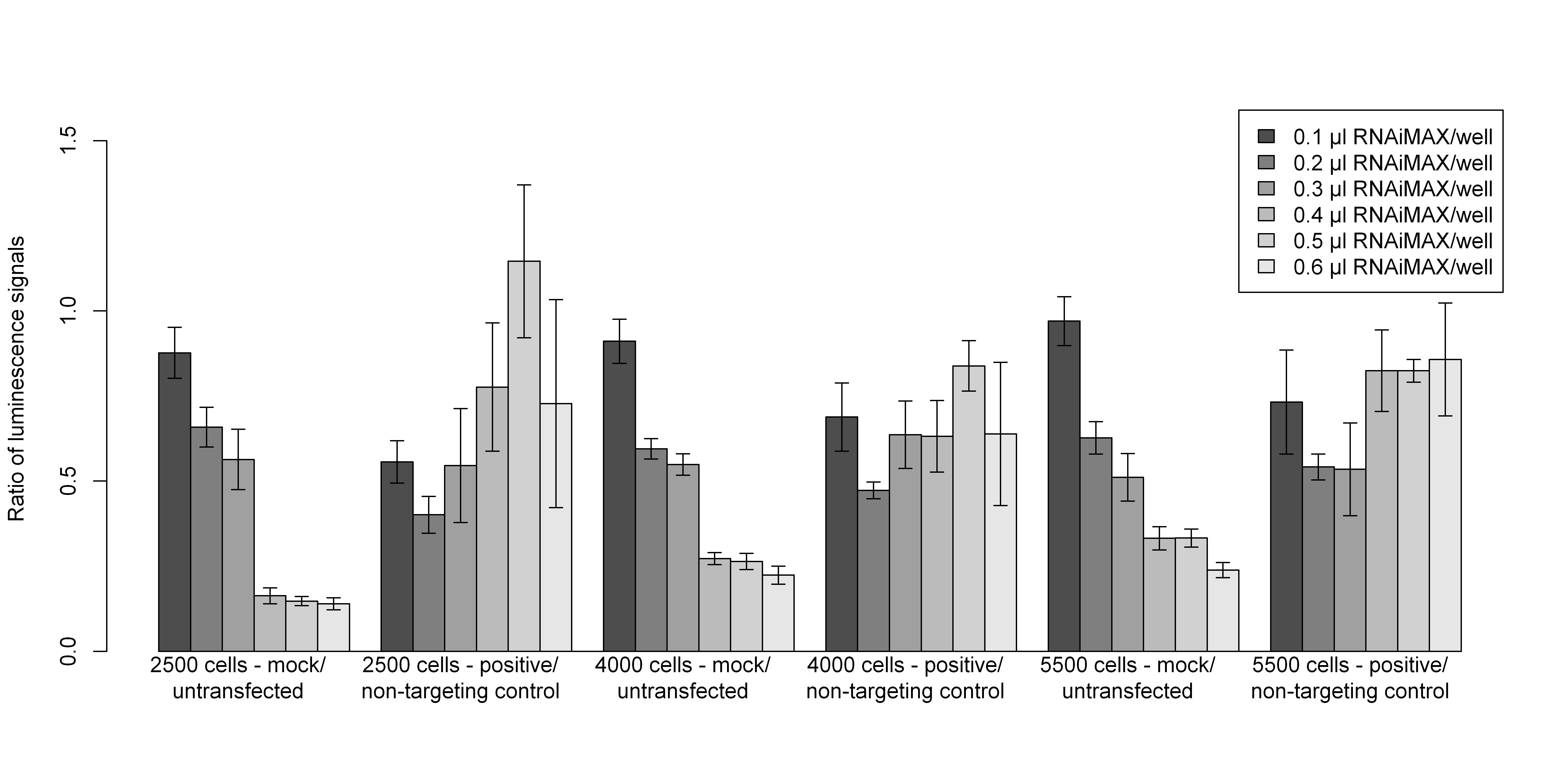
Supplementary Figure 1: Screening assay development.** Ratios of average luminescence signals between mock (n=5 wells per RNAiMAX amount and cell number) and untransfected (0 µl RNAiMAX/well, n=6 wells per cell number), and between cells transfected with a positive (n=5 wells per RNAiMAX amount and cell number) and a non-targeting control (n=5 wells per RNAiMAX amount and cell number) for different cell numbers and amounts of transfection agent. Error bars represent +/- standard deviation.

**
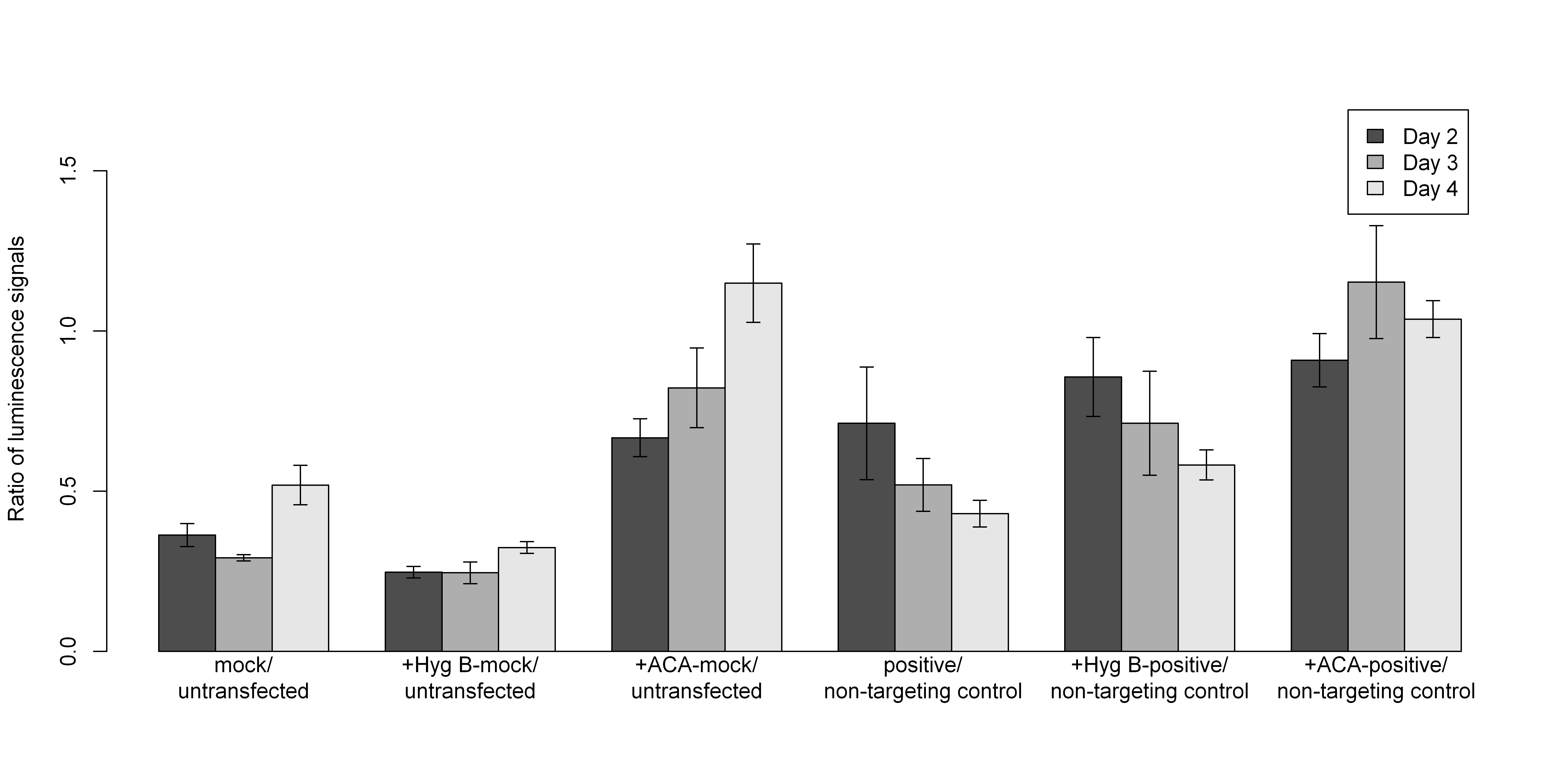
Supplementary Figure 2: Incubation time and media supplement testing.** Ratios of average luminescence signals between mock (n=5 wells per day and supplement (none, +Hyg B: Hygromycin B Gold, +ACA: Anti-clumping agent) and untransfected (0 µl RNAiMAX/well, n=9 wells per day), and between cells transfected with a positive (n=5 wells per day and supplement) and a non-targeting control (n=5 wells per day and supplement) for different time points and media supplements. Error bars represent +/- standard deviation.

**
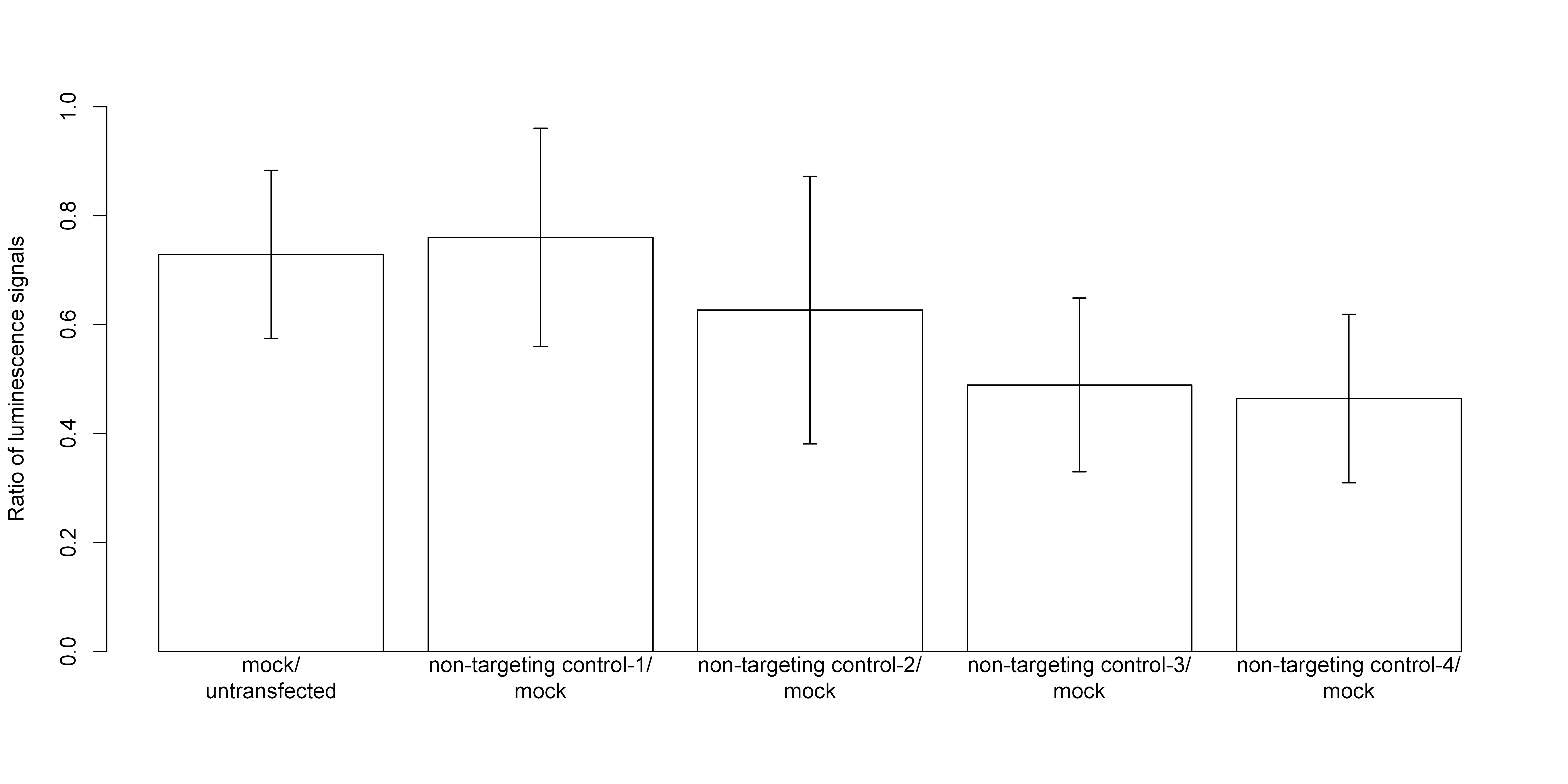
Supplementary Figure 3: Non-targeting control selection.** Ratios of average luminescence signals between mock (n=15 wells) and untransfected (0 µl RNAiMAX/well , n=5 wells), and between cells transfected with different non-targeting siRNAs (n=15 wells per non-targeting control) and the mock control (n=15 wells). Non-targeting 1: Silencer® Select Negative Control No. 1 siRNA. Non-targeting 2: Silencer® Select Negative Control No. 2 siRNA. Non-targeting 3: AllStars Negative Control siRNA. Non-targeting 4: Silencer® Negative Control No. 5 siRNA. Error bars represent +/- standard deviation.

**
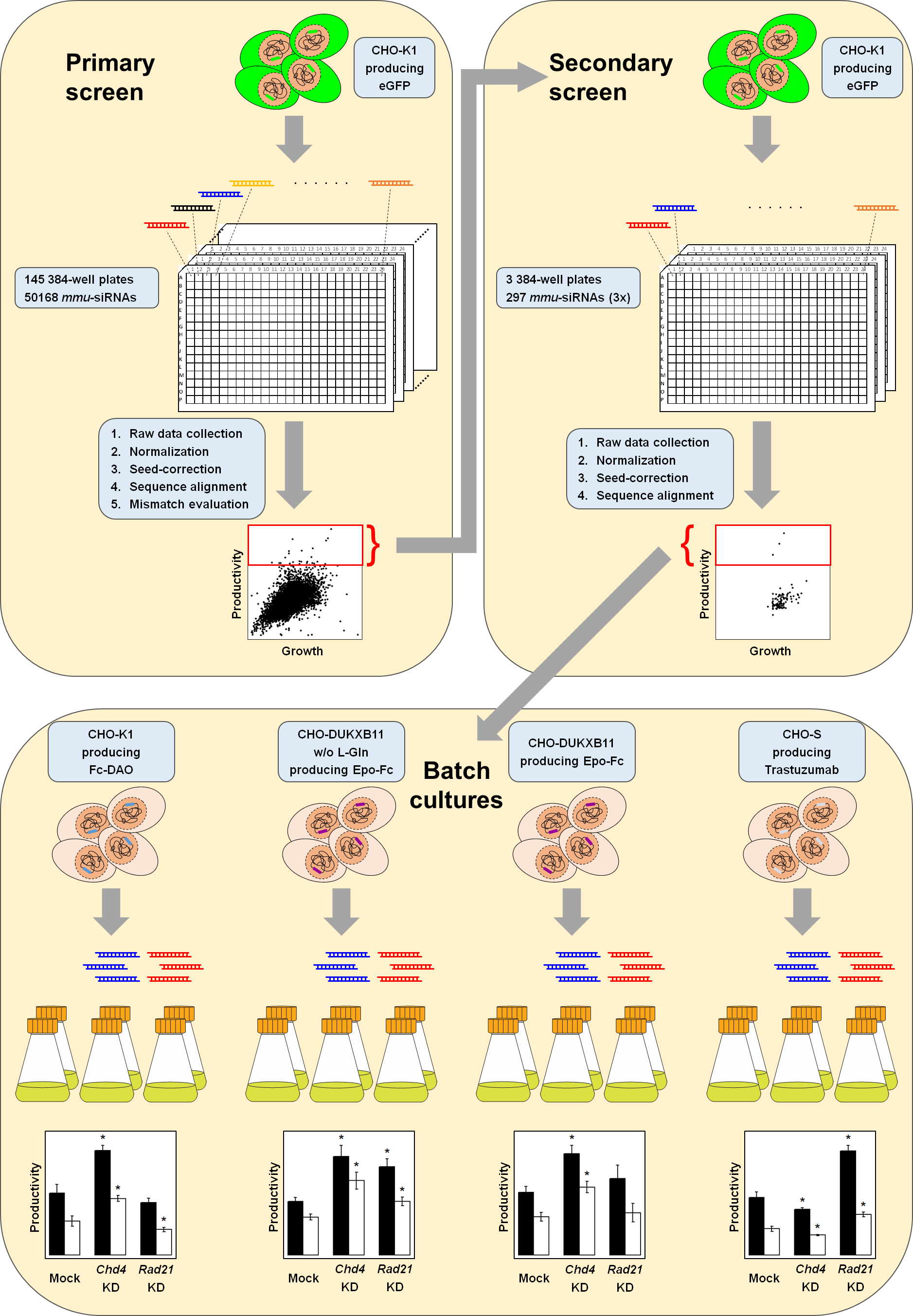
**

**Supplementary Figure 4: Workflow.** Primary screen: A whole genome mouse siRNA library was applied to CHO cells producing eGFP. The collected fluorescence and viable cell density data were normalized and seed-based corrected. Then the siRNA sequences were aligned to available CHO transcriptomes to identify valid siRNAs (perfect alignment). siRNAs with one mismatch against a transcript were evaluated by comparing their effect on the viable cell density from proteasome-associated genes to siRNAs targeting the same genes with a perfect alignment. Secondary screen: Genes with the highest productivity of the primary screen were validated by a second screen, but with each siRNA tested thrice. Batch cultures: Identified and validated hits (*Rad21* and *Chd4*) were tested again in additional cell lines in shaker tubes, and their effect on the productivity was analyzed. Colors of siRNAs indicate different gene targets.

**Supplementary Figure 5: Plate layout of high throughput screen.** Blue: Whole genome library against mouse genome, one siRNA species per well. Green: Non-targeting control. Red: Positive control.

**
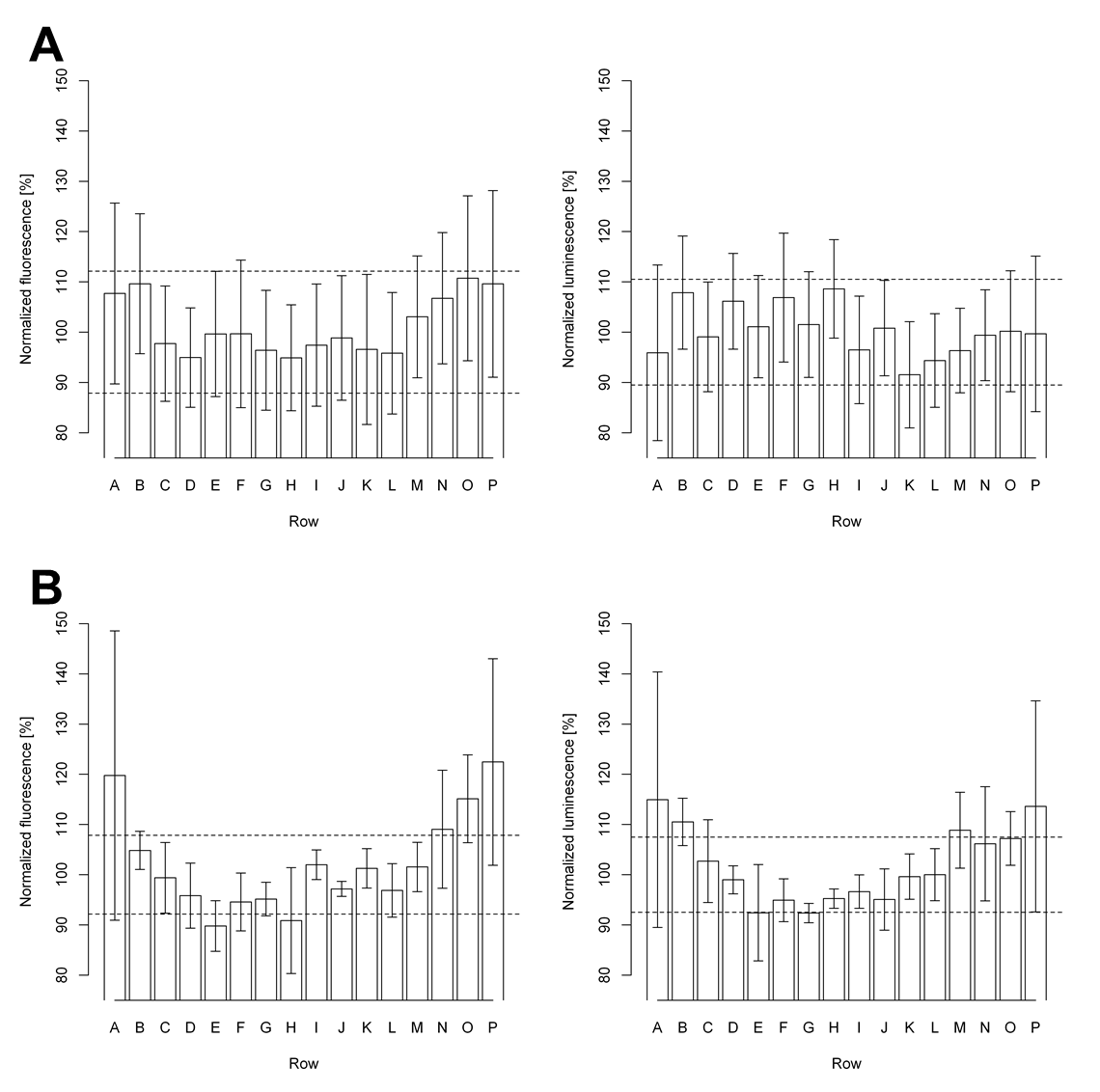
Supplementary Figure 6: Spatial effects.** Median of fluorescence and luminescence measurements for each position of the non-targeting control column of the primary (A, n=145 plates) and secondary (B, n=3 plates) screen. Error bars represent standard deviations of each position. Dashed lines show median of the negative control of the screen +/- 1x Median absolute deviation.

**
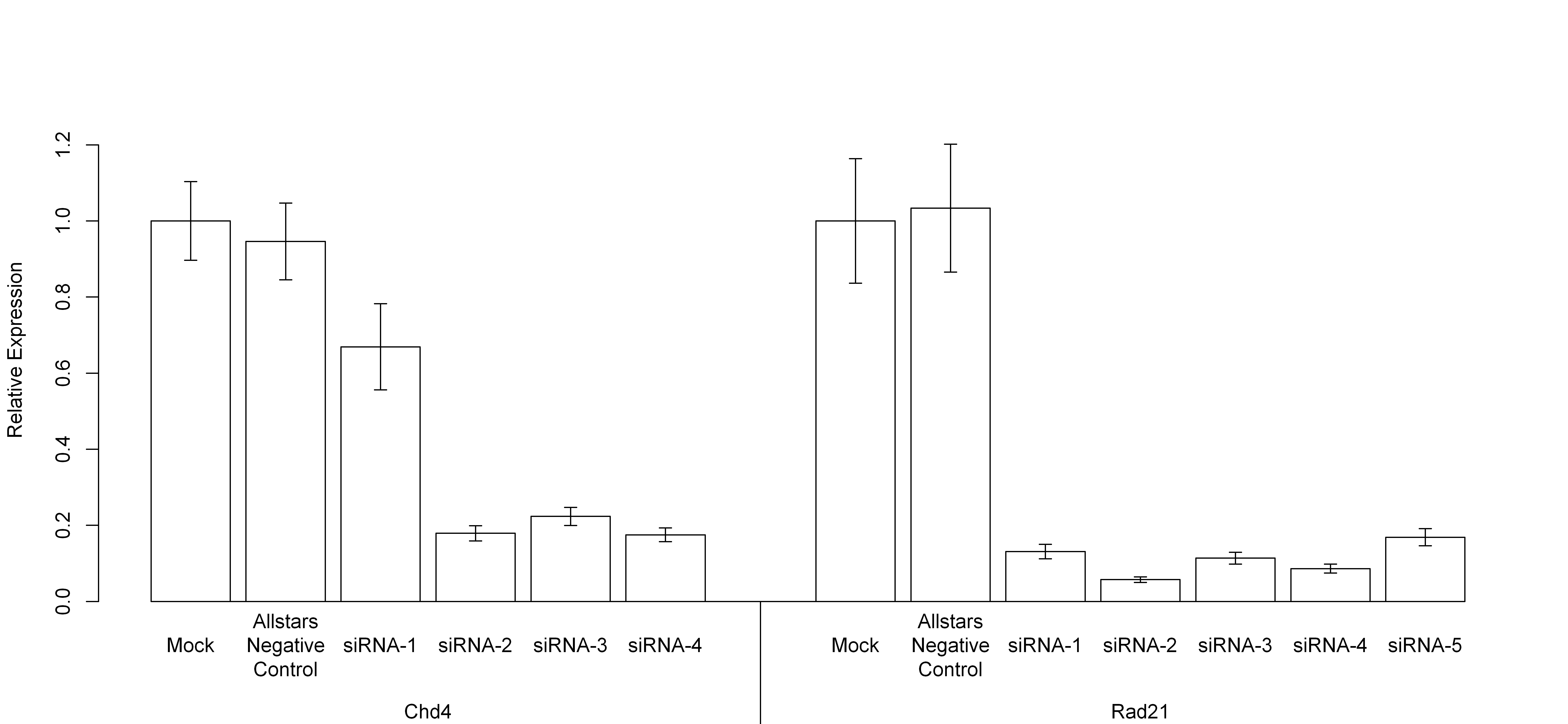
Supplementary Figure 7: Efficiency of different siRNAs targeting *Chd4* or *Rad21*.** Efficiency of four (*Chd4*) or five (*Rad21*) different siRNAs to knock down the respective gene. Bars show the relative expression in comparison to the mock transfection (set to 1) according to the 2^-ΔΔC^_T_ method (n=4 qPCR reactions per sample). Error bars represent +/- standard deviation.
